# Challenges in HIV-Negative Cytomegalovirus Retinitis – case report

**DOI:** 10.22336/rjo.2021.49

**Published:** 2021

**Authors:** Mihail Zemba, Roxana-Elena Rogoz, Alexandra Cătălina Zaharia, Andreea Elena Dimirache, Otilia-Maria Dumitrescu, Diana-Maria Dărăbuş

**Affiliations:** * Ophthalmology Department, “Dr. Carol Davila” Central Military Emergency University Hospital, Bucharest, Romania; ** “Carol Davila” University of Medicine and Farmacy Bucharest, Romania; *** Department of Ophthalmology, “Victor Babeş” University of Medicine and Pharmacy, Timişoara, Romania

**Keywords:** Cytomegalovirus retinitis, HIV seronegative, combined seronegative immunodeficiency, cerebroretinal microangiopathy, Coats’ plus disease

## Abstract

We present the case of a 20-year-old girl with severe combined seronegative immunodeficiency who developed a bilateral decrease in visual acuity due to retinal necrosis. After further investigations, increased serological viral levels of Cytomegalovirus (CMV) were detected and confirmed the diagnosis of CMV retinitis in both eyes. After three weeks of systemic therapy with oral valganciclovir, her condition improved, with the best corrected visual acuity of the most affected eye changing from finger counting at presentation to 6/ 12. Although financial matters determined her to discontinue the antiviral treatment after three months, her ophthalmological status remained stable, and she resumed therapy after four weeks of pause. At the four months follow-up, despite an unchanged visual function, her general condition deteriorated. In the absence of appropriate treatment for her immunodeficiency both the patient’s ophthalmological and systemic prognosis were poor.

## Introduction

Human Cytomegalovirus (CMV), also known as Human Herpes Virus - 5 (HHV-5) - is a ubiquitous virus belonging to the Herpesviridae family. It is frequently encountered worldwide, the prevalence of the infection with CMV reaching almost 100% in Africa and Asia and nearly 80% in North America and Europe [**1**]. Most infections progress asymptomatically and, following primoinfection, a balance develops between the virus and the host’s immune system, a state called latency, and, as such, reactivation is rare in immunocompetent hosts [**2**]. However, CMV infection in immunocompromised patients can have devastating outcomes, with increased morbidity and mortality rates [**3**].

CMV retinitis is an important opportunistic infection occurring in hosts with poor immunological defense mechanisms, most of the cases being reported in Human Immunodeficiency Virus (HIV)-infected patients with a CD4+ lymphocyte T count of 50 cells/ µL or less [**3**]. Less commonly, CMV retinitis is associated with other immunocompromised states, such as in the case of hematological malignancies, iatrogenically-induced immunosuppression following organ transplantation, autoimmune diseases and, sometimes, after treatment with locally administered steroids [**4**,**5**]. However, following the introduction of Highly Active Antiretroviral Therapy (HAART), HIV-related CMV retinitis have begun to decline in their numbers, while more and more HIV-negative cases are being reported [**6**].

Classically, the clinical picture in HIV-positive patients is divided into two disease patterns: a fulminant form, characterized by extensive hemorrhages along the vascular arcades and confluent opaque foci of full-thickness retinal involvement, and an indolent form, with granular opacification and less hemorrhages. A third, perivascular form with a “frosted branch angiitis” aspect has also been described [**7**]. The minimal or absent intraocular inflammation Typical for CMV retinitis in HIV-positive patients [**8**]. However, in HIV-negative subjects, CMV retinitis is associated with more prominent intraocular inflammation and vitritis and a tendency towards retinal arteries involvement, with arteritis and an occlusive pattern [**4**].

The diagnosis is, usually, clinical, based on the funduscopic retinal findings in the context of systemic CMV infection (CMV antibodies and viral load in the peripheral blood serum) and can be further confirmed by performing a vitreous or aqueous tap with subsequent polymerase chain reaction (PCR) identification of CMV deoxyribonucleic acid (DNA) [**7**,**9**]. 

Treatment usually consists in the administration of specific drugs (ganciclovir, valganciclovir, cidofovir or foscarnet) either systemically, intravitreally or a combination of the two [**4**,**7**]. Visual prognosis is often poor, the disease course being marked by recurrences and complications, such as retinal detachment and vascular complications [**7**,**9**]. 

## Case report

**Fig. 1:**
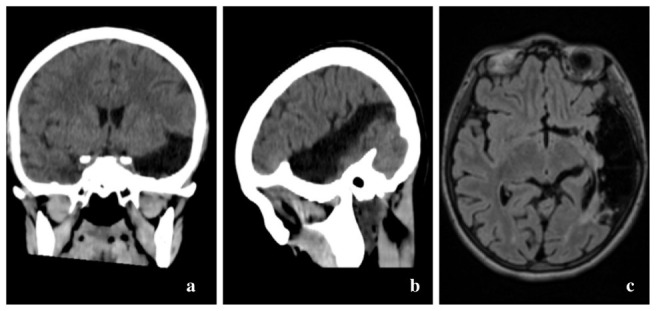
Cranio-cerebral imaging: native computed tomography (CT) in coronal (a) and sagittal (b) sections and magnetic resonance imaging (MRI) in axial section (c) – all images illustrate left parietal-temporal encephalomalacia

We present the case of a 20-year-old female with mild mental disability who was referred to us for ophthalmic evaluation from another medical department, as she complained of blurred vision in both eyes, but more prominent in the right eye, that had appeared two weeks prior to presentation. From the patient’s medical history, it is essential to mention a severe combined immunodeficiency disease for which the patient received treatment consisting of immunoglobulin substitution therapy, prophylactic antibiotics (trimethoprim/ sulfamethoxazole), antivirals (Acyclovir) and antimycotic medication (Fluconazole). Test results for HIV were negative at the time. Other important disorders with impact on the long-term prognosis were a left middle cerebral artery stroke of a superficial territory, with left parietal-temporal encephalomalacia (**Fig. 1**) and pulmonary involvement comprising bronchiectasis and pulmonary nodules. Also, genetic testing dating from 2018 revealed a mutation in TTC7A NM_020458 gene: exon 8:c.G1027A;p.E343K (homozygous).

**Fig. 2:**
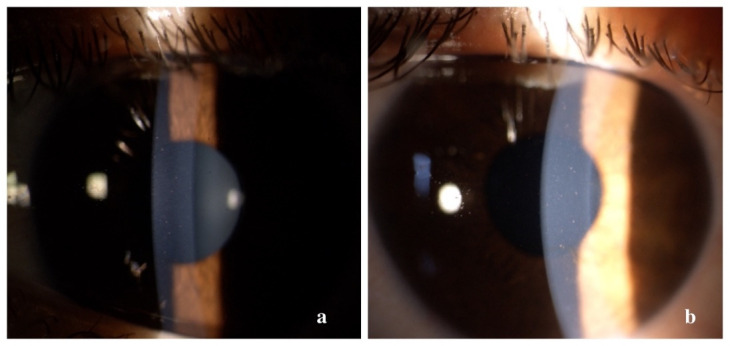
Slit lamp image of stellate keratic precipitates (a - right eye, b - left eye)

At presentation, the patient’s best corrected visual acuity (BCVA) was finger counting in the right eye, with light perception only from the temporal and the inferior aspects, and 6/ 12 in the left eye. A relative afferent pupillary defect (RAPD) was present in the right eye and was more subtle in the left eye. Intraocular pressure was within normal limits. On slit lamp examination, stellate keratic precipitates were noted in both eyes (**Fig. 2**).

**Fig. 3:**
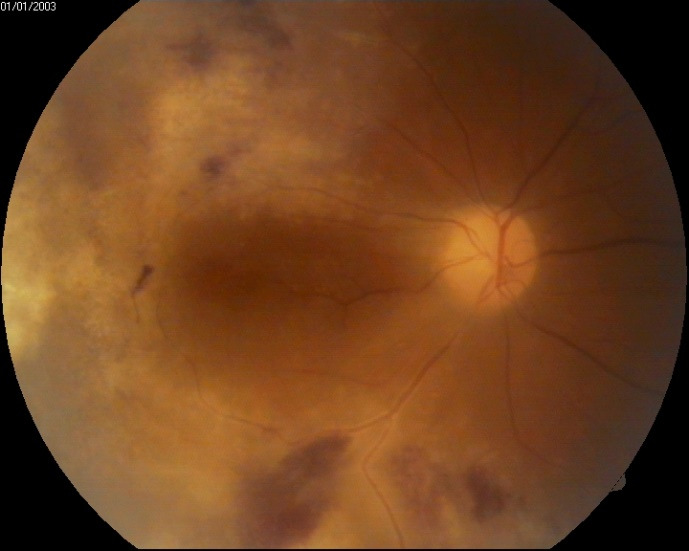
Right eye fundus at presentation: blurred temporal papillary contour, cloudy yellow-white retinal lesions, hemorrhages, and cotton-wool spots – “pizza pie” appearance

**Fig. 4:**
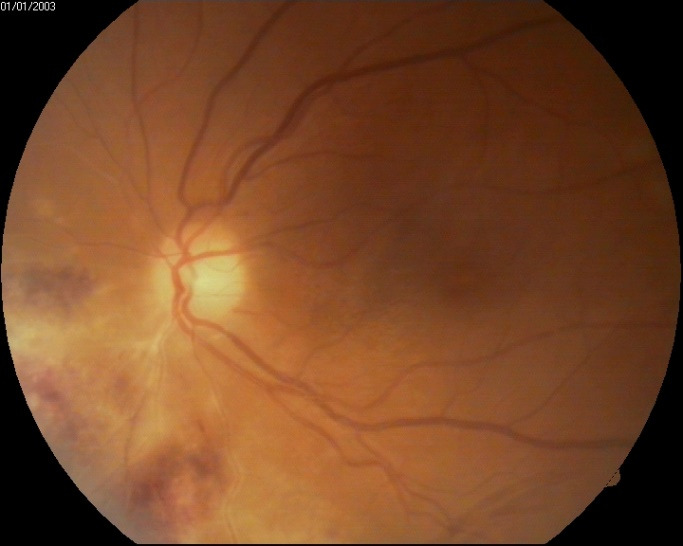
Left eye fundus at presentation: normal papillary aspect, retinal lesions, and hemorrhages nasally

Fundoscopic evaluation of the optic disc revealed no papilledema, no changes in shape or cupping, but only a slight discoloration of the disc, as well as a blurred temporal contour in the right eye, while a normal aspect was noted in the left eye. Multiple retinal changes were present, including diffuse, large yellow-white cloudy retinal lesions, hemorrhages, and cotton wool spots, a “pizza-pie” aspect suggestive of CMV retinitis. The changes were more extended in the right eye and limited to the inferior temporal and nasal retina in the left eye and did not appear to involve the macula, although the central region was slightly elevated, with an absent foveal reflex (**Fig. 3**, **4**). Moreover, a grade 1 vitritis was noted in right eye.

**Fig. 5:**
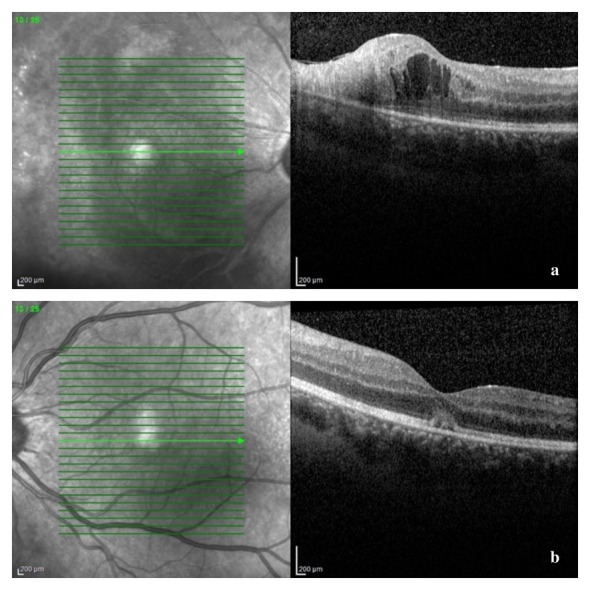
SD-OCT of macular region at presentation: a – right eye: disruption of the outer retinal layers and macular cystoid oedema; b – left eye: thickening of retinal layers and a vitelliform-like deposit

**Fig. 6:**
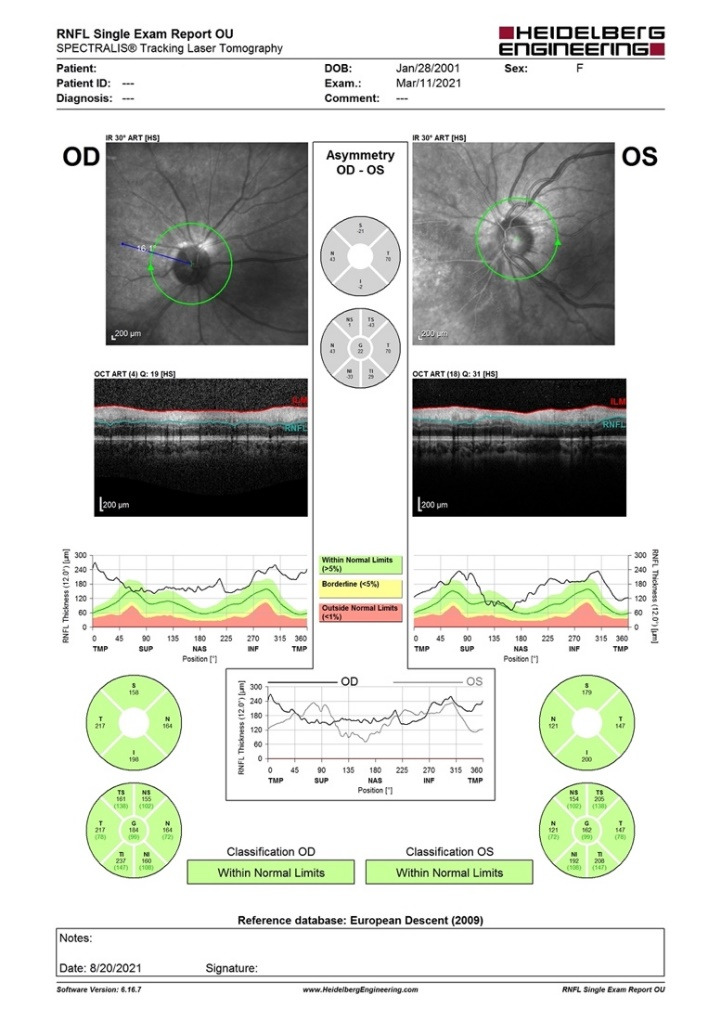
SD-OCT of the optic nerve: thickening of the retinal nerve fiber layer

A B-scan ultrasound was performed and confirmed the presence of vitritis, showing mild dense, slightly clumped vitreous opacities, without retinal detachment. The Spectral Domain Optical Coherence Tomography (SD-OCT) confirmed our suspicion of macular oedema, showing intraretinal fluid and cystic spaces (**Fig. 5**). The optic nerve appeared thickened in both eyes, suggesting a subclinical nerve fiber oedema (**Fig. 6**). The visual field could not be obtained at presentation due to her clinical status and lack of cooperation.

Considering the patient’s severe immunodeficiency and the clinical aspect, the initial presumptive diagnosis was CMV retinitis in both eyes. We recommended serological testing for both CMV and Herpes Simplex Virus (HSV), in order to rule out a possible acute retinal necrosis, in which the etiological agent is usually either HSV or Varicella Zoster Virus and only very rarely CMV.

Immunological testing showed lymphocytopenia with deficiencies in all three (T, B and NK) cell lines. Serological testing revealed a high CMV viral load of 4294 UI/ ml (normal value < 178 UI/ ml), while the HSV and the HIV RNA testing were negative. Laboratory results confirmed the diagnosis of CMV retinitis in both eyes in a seronegative patient. Treatment with valganciclovir 900 mg twice daily was initiated. After one week of therapy, our patient’s visual acuity had improved in the right eye to 6/ 15 and to 6/ 12 in the left eye. The clinical aspect was similar to the previous examination in both eyes.

**Fig. 7:**
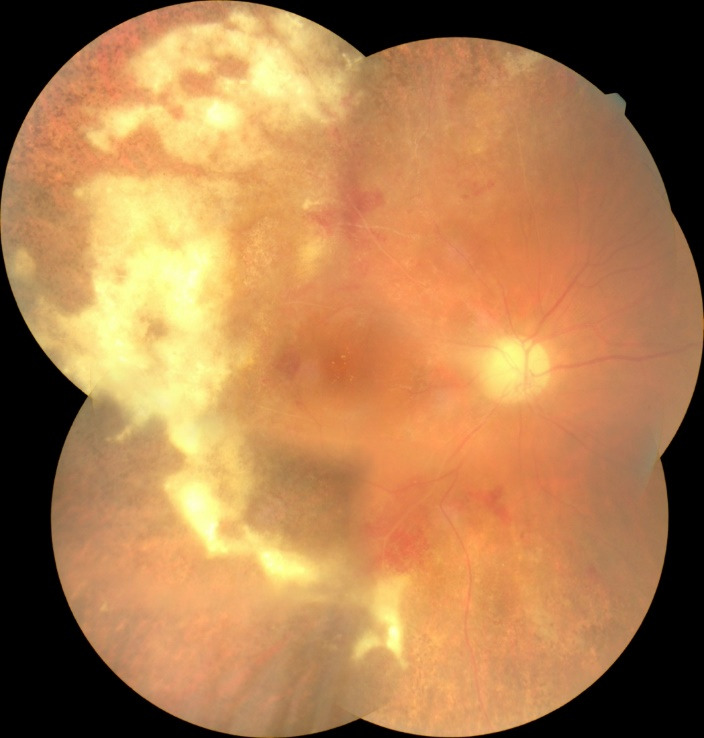
Fundoscopic aspect of the right eye after one month of therapy: macular hard exudates, resorption of retinal hemorrhages and temporal retinal atrophy

We reevaluated the patient after one and three weeks and then after one month of treatment. Her BCVA had improved to 6/ 12 in right eye and 6/ 10 in left eye, with normal RAPD, normal intraocular pressure, and normal anterior segment aspect, at that moment. Fundus examination revealed resorption of retinal hemorrhages, less extended retinal atrophy, and macular hard exudates, alongside unperfused vessels from the temporal arcades in right eye and nasal vessels in the left eye. There were no signs of disease progression (**Fig. 7**, **8** at one month). SD-OCT scans showed the persistence of the diffuse retinal layer oedema, a central minimal neuroepithelium detachment and fibrosis of the temporal retina in the right eye, while in left eye retinal layers retained an increased thickness and the vitelliform-like deposit persisted (**Fig. 9** at one week and **Fig. 10** at three weeks). We performed a Humphrey static automated perimetry, which revealed a diffuse reduction in retinal sensitivity in both eyes. In the right eye, this reduction was more profound and there were extended areas of complete loss of sensitivity in the nasal field.

**Fig. 8:**
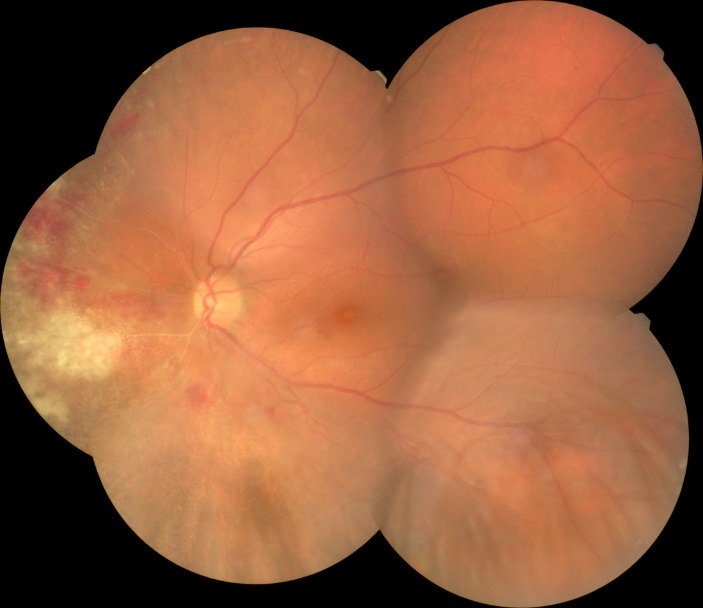
Fundoscopic aspect of the left eye after one month of therapy: vitelliform-like macular lesion, partial resorption of retinal hemorrhages, nasal unperfused blood vessel and retinal atrophy

**Fig. 9:**
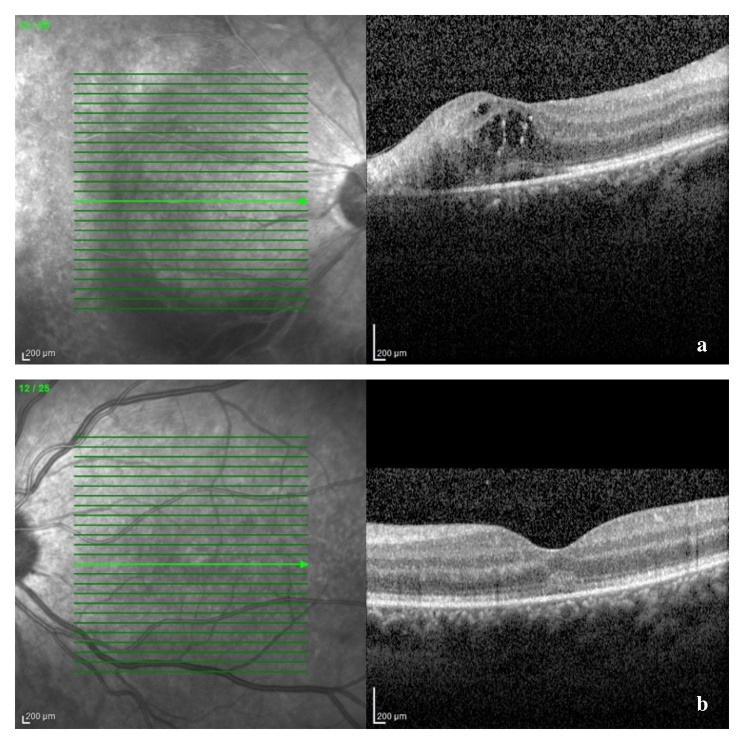
SD-OCT of the macular region after one week of therapy: a - right eye: amelioration of the structural alteration of the temporal macular layers, with central cystoid oedema; b – left eye: reduction of the vitelliform-like deposit

**Fig. 10:**
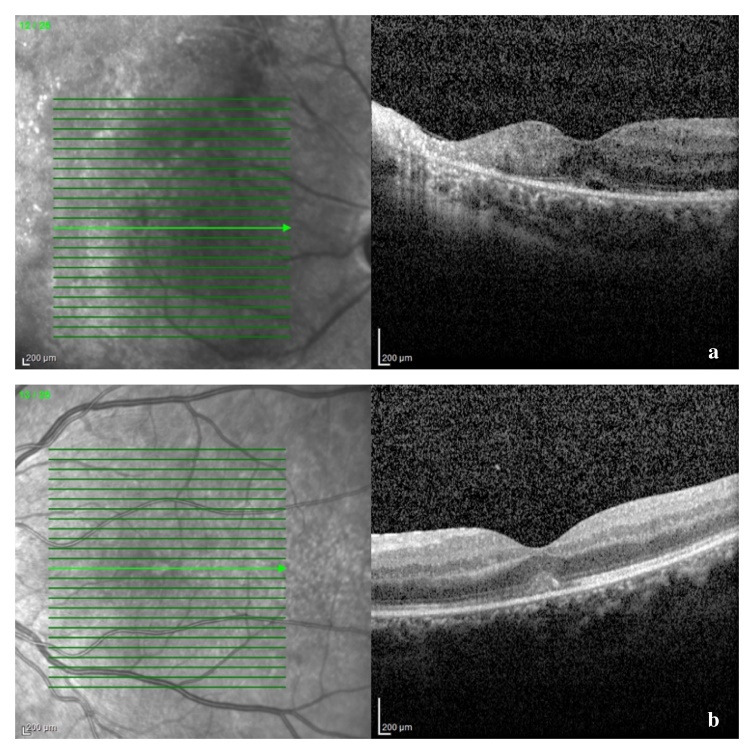
SD-OCT of the macular area after three weeks of therapy: a – right eye: parafoveal temporal hyperreflective fibrous tissue (retinal atrophy) and minimal central neuroepithelium detachment

**Fig. 11:**
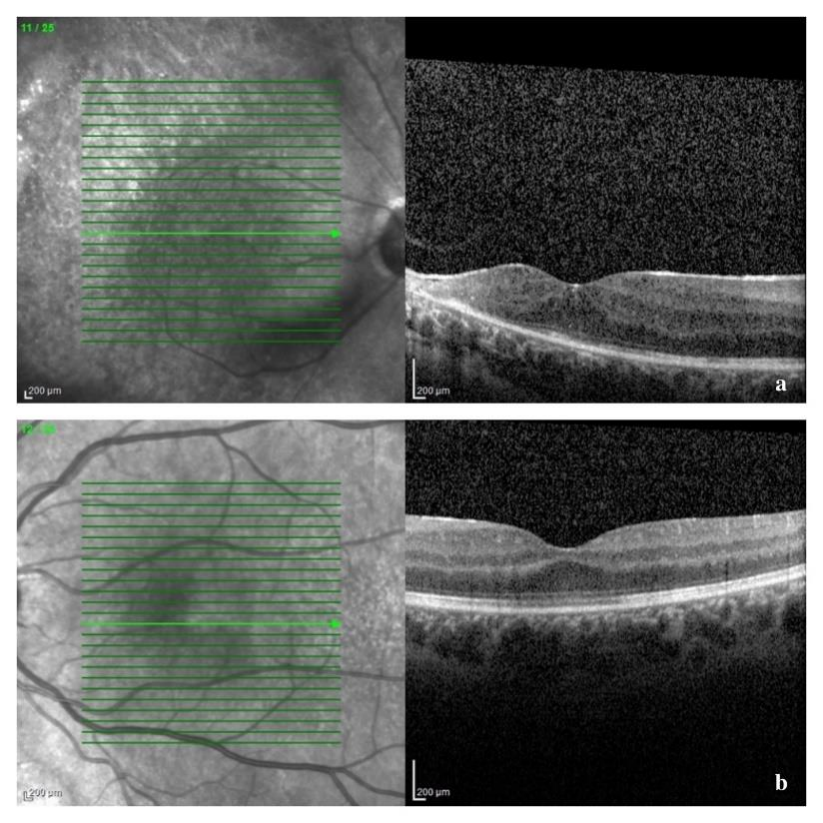
SD-OCT of the macular area after two months of treatment: a – right eye: resorption of hard exudates; b – left eye: resorption of the vitelliform-like deposit

Due to an overall positive response to treatment, we recommended a minimum of six months of therapy in order to prevent a relapse, along with monthly reexamination. The follow-ups at two and three months after the initiation of therapy revealed similar ophthalmological findings, with no active disease (**Fig. 11**). However, due to financial issues, the patient had discontinued the antiviral treatment for four weeks, so at the three months check-up she was no longer under therapy. Even in the absence of the systemic antiviral therapy, her ophthalmological status remained stable.

**Fig. 12:**
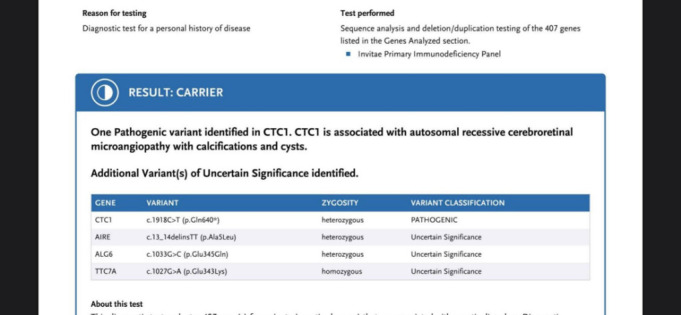
Genetic testing results: heterozygous for the CTC1, AIRE and ALG6 mutations and homozygous for the TTC7A mutation

She resumed oral antiviral therapy and at the 4-month reexamination, her visual status remained unchanged, with the same VA of 6/ 12 in the right eye and 6/ 10 in the left eye. However, her general condition deteriorated, as she complained of pain in left arm and leg and cooperation was poor. Her physician from the internal medicine department recommended a genetic testing including 407 genes. Results revealed a mutation in conserved telomere maintenance component 1 (CTC1) gene, unknown before (**Fig. 12**).

## Discussions

Most HIV-unrelated cases of CMV retinitis appear in the setting of hematological malignancies or in patients requiring systemic immunosuppression, such as after organ transplantation or in autoimmune diseases [**9**]. Our case owes its most important particularity to the background diagnosis and cause of immunosuppression, namely the severe combined immunodeficiency syndrome (SCID). SCID is a form of primary immunodeficiency characterized by marked lymphocytopenia (CD3+ cells < 300/ µl), although there are atypical forms with CD3+ cell numbers of > 300/ µl, associated with functional impairment of T cells. Moreover, in certain subgroups of patients, there is an accompanying quantitative deficit of B cells and/ or natural-killer (NK) cells. Our patient had deficiencies of all three (T, B and NK) cell lines. Typically, in the absence of a bone marrow stem cell transplant, affected infants die of severe infections within the first two years of life, although particular mutations, which allow for some production of functional T cells, may allow survival until adulthood [**10**]. Our patient had a mutation in the TTC7A gene, which is amongst the most rare to be associated with SCID [**11**]. There are few reports of the association between CMV retinitis and SCID and none, to our knowledge, in a patient having reached early adulthood. 

Clinical evaluation is the cornerstone of diagnosis. Bilateral involvement is common, either at presentation or developing during the following weeks [**4**,**9**,**12**]. Although there are studies that report a similar clinical picture for CMV retinitis in HIV-negative patients compared to the classically described HIV-related forms [**12**,**13**], there have been reports of certain features that are more commonly associated with the former [**4**,**9**,**12**]. Ho et al. studied the particularities of CMV retinitis in HIV-negative versus HIV-positive immunocompromised patients and found that a vascular occlusive pattern (p = 0.003), retinal arteritis (p = 0.016) and prominent vitritis (p =0.04) were significantly more frequent in the HIV-negative group [**4**]. These particularities may be the result of the different type and level of immunosuppression associated with HIV-negative cases, which may explain the more prominent ocular inflammation found in these patients and the ensuing vitritis and retinal vasculitis. What is more, in cases of HIV-negative patients with less severe immunodepression, Schneider et al. described a variant that mimicked acute retinal necrosis, but which followed a more indolent, subacute or chronic, course, suggesting the diagnosis of CMV retinitis [**14**]. Some of the above traits are present in our patient, which displayed a fulminant form of retinitis, especially in the right eye, with widespread retinal involvement (more than two quadrants) including zone I (defined as the area located within 3 mm from the fovea or within 1.5 mm from the optic nerve), as well as retinal vasculitis. Vitritis was minimal, probably as a result of the low T cell count. Iu et al. [**9**] and Vishnevskia-Dai et al. [**15**] reported concurrent anterior segment involvement, most frequently with anterior chamber cells and keratic precipitates. In our case, the patient had bilateral diffuse non-granulomatous keratic precipitates, that later improved with therapy. 

The definite diagnosis was based on the clinical examination in the context of elevated blood serum viral load (4294 IU/ ml), which indicated an active systemic CMV infection. Other authors found detection of viral DNA in the aqueous humor or in the vitreous using PCR to be more sensitive [**4**], but this was considered unnecessary in our case, as the clinical picture together with the viral load were enough to make the diagnosis. 

Following diagnosis, our patient received specific systemic anti-viral treatment (oral valganciclovir 900 mg daily). Martin et al. proved in their study the non-inferiority of oral valganciclovir over intravenous ganciclovir both in the induction and the maintenance phases of treatment [**16**]. Systemic therapy may be associated with intravitreal antiviral therapy (ganciclovir, foscarnet or cidofovir), especially in cases in which the response to systemic therapy is insufficient, where there is sight-threatening involvement or when the toxicity of the systemic treatment prevents its administration [**7**,**13**,**15**]. Nevertheless, Ho and colleagues [**4**] found that the use of intravitreal anti-CMV therapy did not influence the final VA. What is more, repeated intraocular injections are associated with an increased risk of retinal vascular occlusion, including macular infarction [**4**], and, also, of retinal detachment, a sight-threatening complication which already has a high incidence, of 3% to 8.7% per year [**4**,**12**,**17**], in HIV-negative patients with CMV retinitis. Our patient improved considerably after 2 weeks of systemic treatment, showing not only lack of progression, but even a slight improvement of retinal lesions, a reduction in the number of keratic precipitates, an amelioration of the macular edema on SD-OCT and, most importantly, an increase in BCVA from finger counting to 6/ 12 in the right eye and a constant BCVA of 6/ 10 in the left eye. Treatment may need to be administered for a long period of time, of months and even years, in order to prevent recurrences [**4**]. We also considered intravitreal injections with Triamcinolone in order to reduce the cystoid macular oedema. However, as glucocorticoids decrease local immunity, their use in the setting of viral infections is debatable and the patient’s infectious diseases physician advised against their use. Moreover, anti-VEGF drugs benefits are questionable, considering our patient’s macular oedema was recent and is not related to the presence of abnormal retinal vessels. Recurrences appear to be related to the discontinuation of the anti-viral medication [**9**]. Cessation of specific therapy is usually dictated by the appearance of the retinal lesions, which should be inactive (retinal scarring) at the time of therapy discontinuation [**7**,**9**]. Qian et al. [**17**] found that the required length of therapy and the initial CMV DNA load in the aqueous humor had a positive correlation. Progression of the disease despite adequate anti-viral therapy can occur in patients with severe immunosuppression. As such, efforts to improve immune status or to adjust immunosuppression therapy are essential for improving prognosis [**4**,**12**]. Our patient maintained a stable improvement despite temporary discontinuation of therapy. 

The clinical course and visual prognosis appears to be independent from the HIV infection status [**4**,**12**,**13**]. Visual prognosis is often poor, with less than half of the patients retaining a VA of more than 20/ 70 [**9**,**12**], while Iu et al. report a proportion as high as 25% of patients with a final VA of less than 20/ 400 [**9**]. The most important factors that appear to significantly affect the visual outcome are the initial VA [**4**,**9**], zone 1 involvement [**13**], the dimension of the retinal lesion [**17**], retinal detachment complicating the course of the disease [**13**] and poor general health [**13**]. The presence of macular involvement in particular was associated with a final VA of less than 20/ 400 [**9**]. Our patient regained a satisfactory BCVA of 6/ 12 in the most affected eye at the last follow-up visit, at 16 weeks after the first presentation, despite the initial BCVA of counting fingers. However, further observation and continuation of the anti-viral treatment is warranted. 

Another aspect that makes this case worthy of note is its complexity, which created the need for a close collaboration between multiple medical departments, all in the setting of a difficult collaboration with the patient. During her follow-up visits in the internal medicine department, another mutation, one in the conserved telomere maintenance component 1 (CTC1) gene, was discovered. This mutation produces telomere dysfunction with instability of STN1, a protein coding gene, and reduced ability to interact with DNA, generating cellular proliferation disturbance. The mutation is also assumed to be associated with autosomal recessive cerebroretinal microangiopathy (Coats’ plus disease), with calcifications and cysts, which could explain the patient’s neurologic and retinal inadequacy, microangiopathy with abnormal vascular permeability, calcifications and cysts formation [**18**]. Moreover, the cerebral and pulmonary lesions have shown signs of progression, consistent with the worsening of symptoms. As such, while the patient’s visual function is currently satisfactory and stable, the overall prognosis is deeply unfavorable and she must be kept under close monitoring in order to promptly detect any life-threatening complications. 

## Conclusions

The complexity of the case and the association of CMV retinitis with this rare disease (Coat’s plus disease) represented a challenge for all the physicians who have treated this patient. Our case also stands as a proof that systemic antiviral therapy has an important role in the treatment of CMV retinitis, improving the symptoms, the visual function, and the clinical appearance.

**Conflict of Interest statement**

The authors state no conflict of interest.

**Informed Consent and Human and Animal Rights statement**

Informed consent has been obtained from all individuals included in this study.

**Authorization for the use of human subjects**

Ethical approval: The research related to human use complies with all the relevant national regulations, institutional policies, is in accordance with the tenets of the Helsinki Declaration, and has been approved by the review board of “Dr. Carol Davila” Central Military Emergency University Hospital, Bucharest, Romania.

**Acknowledgements**

None.

**Sources of Funding**

None.

**Disclosures**

None.
